# Physician Awareness and Utilization of Food and Drug Administration (FDA)-Approved Labeling for Pharmacogenomic Testing Information

**DOI:** 10.3390/jpm3020111

**Published:** 2013-06-10

**Authors:** Eric J. Stanek, Christopher L. Sanders, Felix W. Frueh

**Affiliations:** 1 Medco Research Institute, LLC, Medco Health Solutions, Inc., Bethesda, MD 20814, USA; E-Mails: csanders@biocerna.com (C.L.S.); felix@frueh.us (F.W.F.); 2 EJS Health Solutions, LLC, 1653 Pennfield Drive Thorofare, West Deptford, NJ 08086, USA; 3 Biocerna, LLC, Gaithersburg, MD 20879, USA; 4 Opus Three, LLC, Gaithersburg, MD 20878, USA

**Keywords:** pharmacogenomics, physician, drug labeling, survey

## Abstract

We surveyed 10,303 United States physicians on where they obtain pharmacogenomic testing information. Thirty-nine percent indicated that they obtained this from drug labeling. Factors positively associated with this response included older age, postgraduate instruction, using other information sources, regulatory approval/ recommendation of testing, reliance on labeling for information, and perception that patients have benefited from testing. Physicians use pharmacogenomic testing information from drug labeling, highlighting the importance of labeling information that is conducive to practice application.

## 1. Introduction

Pharmacogenomics is a rapidly evolving field, and it is increasingly challenging for physicians and other healthcare practitioners to keep abreast of new information on pharmacogenomic testing that impacts the prescription and clinical monitoring of drug therapy. Recent surveys have indicated that physicians are in need of education, and reliable and accessible information sources regarding pharmacogenomics and related testing [1,2,3]. United States Food and Drug Administration (FDA)-approved labeling is a primary source of drug information. The number of drugs with pharmacogenomic information in their labeling, in particular that of actionable nature, and the number of patients prescribed these drugs, have dramatically expanded over the past several years [4,5,6]. However, the extent to which physicians may actually use or value FDA-approved drug labeling or other sources of pharmacogenomic testing information remains largely unknown. 

The objective of this study was to establish an initial measure of the proportion of United States-based physicians who obtain pharmacogenomic testing information from drug labeling. Further, this study sought to describe these physicians in terms of their predisposing basic and professional characteristics, as well as their knowledge, training, beliefs, readiness to adopt, concerns, and adoption of pharmacogenomic testing. 

## 2. Results

### 2.1. Survey Performance

Overall survey performance, generalizability, and full results have been reported in-detail elsewhere [1]. In brief, 388,459 physicians were faxed the survey and 10,303 physicians (approximately 3%) returned surveys that met criteria for inclusion in the final analysis. All 10,303 respondents provided a response to the survey item, “Where do you obtain information on genetic testing and its application in the context of drug therapy?” A total of 4,054 physicians (39.4%) indicated drug labeling (package insert) was used as an information source among other nonexclusive response options (Internet; Genetic testing laboratory; Colleague/Other physician; or Other), and served as the main sample for the current analysis. Additionally, 4,184 (41.5%) physicians responded affirmatively to the survey question “Do you rely on FDA-approved labeling (package inserts) for information regarding genetic testing to predict or improve the response to drugs?”

### 2.2. General Assessment of Physicians who obtain Pharmacogenomic Information from Drug Labels Compared to the Entire Survey Sample

Table 1 provides the characteristics of the physicians who reported obtaining pharmacogenomic information from drug labels, and those of the overall survey responder sample for reference. The two groups are generally quite similar, with the exception of a tendency for the physicians who reported obtaining pharmacogenomic information from drug labels to be slightly older, a greater number of years in practice post-graduation, work in private practice, and less often a US medical school graduate. 

Survey responses relating to pharmacogenomics, for the physicians who reported obtaining pharmacogenomic information from drug labels and those of the overall survey responder sample, are displayed in Table 2. In general, physicians who utilized drug labels for pharmacogenomics information were more likely to be educated and feel informed on the subject, have adopted pharmacogenomics in practice, and perceive that pharmacogenomic testing has benefitted their patients in some manner. They were very strongly reliant on FDA labeling for pharmacogenomics information, and more often used other information sources such as the Internet, genetic testing lab, and colleagues/other physicians. When considering whether to order a pharmacogenomic test, they tended to weigh FDA approval/recommendation as a very important/important level of evidence. 

**Table 1:** Physician and practice characteristics.

Characteristic	Obtain PGx Info From FDA Label; N = 4054 N (%)	All survey responders; N = 10303 N (%)
Age (years)		
20–29	16 (0.4)	36 (0.4)
30–39	386 (9.6)	1,085 (10.6)
40–49	755 (18.8)	2,153 (21.1)
50–59	1,372 (34.1)	3,510 (34.3)
60–69	996 (24.8)	2,430 (23.8)
≥70	499 (12.4)	1,013 (9.9)
Gender		
Female	1,158 (29.0)	2,935 (28.9)
Male	2,838 (71.0)	7,233 (71.1)
Medical degree		
MD	3,524 (88.1)	9,040 (88.8)
DO	335 (8.4)	852 (8.4)
Other	141 (3.5)	290 (2.8)
Practice region		
Northeast	917 (22.9)	2,347 (23.0)
South	1,468 (36.6)	3,621 (35.5)
Midwest	895 (22.3)	2,283 (22.4)
West	729 (18.2)	1,936 (19.0)
Years since medical school graduation		
<14	681 (17.0)	1,925 (18.9)
15–29	1,628 (40.7)	4,307 (42.2)
30+	1,695 (42.3)	3,969 (38.9)
Practice specialty		
Family/general practice, internal, preventive	1,540 (39.3)	3,917 (39.2)
Non-surgical specialty	1,975 (50.4)	5,065 (50.7)
Surgical specialty	145 (3.7)	353 (3.5)
Oncology	65 (1.7)	130 (1.3)
Other	192 (4.9)	532 (5.3)
Practice setting		
Rural	807 (20.2)	2,059 (20.3)
Suburban	1,895 (47.4)	4,840 (47.7)
Urban	1,298 (32.5)	3,251 (32.0)
Location of medical school attended		
United States	3,075 (76.8)	8,255 (81.0)
Europe	156 (3.9)	343 (3.4)
Other	772 (19.3)	1,595 (15.7)
Primary employer		
Federal/state/military/Veterans Administration	43 (1.1)	119 (1.2)
Hospital/HMO/other health insurer	296 (7.4)	843 (8.3)
Medical school/university	100 (2.5)	275 (2.7)
Private practice/self-employed	3,445 (86.3)	8,611 (84.8)
Other	110 (2.8)	311 (3.1)
Patient visits per day (on average)		
None	16 (0.4)	37 (0.4)
1–9	550 (5.4)	1,434 (14.1)
10–20	1,728 (43.4)	4,519 (44.5)
21+	1,692 (42.5)	4,157 (41.0)
Primary insurance carrier for majority of patients		
Medicaid	307 (8.1)	778 (8.0)
Medicare	1,222 (32.1)	2,913 (30.0)
Military/Tricare	11 (0.3)	24 (0.3)
Private insurance	2,118 (55.6)	5,554 (57.2)
Veterans Administration	6 (0.2)	20 (0.2)
None	145 (3.8)	426 (4.4)

**Table 2:** Physician knowledge, perception, and practices relating to pharmacogenomics.

Survey question	Obtain PGx Info From FDA Label; N = 4054 N (%)	All Survey Responders; N = 10303 N (%)
Was pharmacogenomics instruction included:		
In your graduate medical education curriculum?	707 (18.0)	3,224 (14.7)
In your postgraduate medical education?	926 (26.9)	2,019 (23.0)
Do you believe that a patient’s genetic profile may influence his/her response to drug therapy?	3,920 (98.0)	9,870 (97.6)
Do you feel that you are adequately informed about the availability of genetic testing and its application in the context of drug therapy?	477(11.9)	1,048(10.3)
At any time in the past 6 months, have you ordered or recommended a pharmacogenomic test?	611 (15.2)	1,319 (12.9)
Do you anticipate ordering or recommending a pharmacogenomic test for a patient within the next 6 months?	1,221 (31.5)	2,592 (26.4)
Where do you obtain information on genetic testing and its application in the context of drug therapy? (select all that apply)		
Internet	1,709 (42.2)	3,519 (34.2)
Genetic testing laboratory	639 (15.8)	1,377 (13.4)
Colleague/other physician	2,036 (50.2)	4,292 (41.7)
Other	824 (20.3)	3,316 (32.2)
Do you rely on FDA-approved labeling (package inserts) for information regarding genetic testing to predict or improve the response to drugs?	3016 (75.4)	4184 (41.5)
Pharmacogenomic tests have benefited your patients by (select all that apply):		
Improving drug effectiveness	490 (12.1)	966 (9.4)
Reducing drug toxicity	542 (13.4)	1,057 (10.3)
Increasing patients’ understanding of their disease/therapy	407 (10.0)	804 (7.8)
Improving patients’ adherence to therapy	216 (5.3)	418 (4.1)
No tests ordered	2,765 (68.2)	7,214 (70.0)
Patients have not benefited	268 (6.6)	824 (8.0)
Are you more concerned about the loss of privacy of a patient’s genetic information from the results of pharmacogenomic tests than from the results of other laboratory or diagnostic tests?	1,277 (32.0)	3,243 (32.0)
Do you believe that private, state, and federal health insurers should provide full coverage for pharmacogenomic tests?		
Always	1,323 (33.5)	3,180 (32.1)
Sometimes	2,440 (61.7)	6,158 (62.1)
Never	192 (4.9)	580 (5.9)
Have you ordered, recommended, or obtained a genome-wide scan for anyone at any time in the past 6 months?	255 (6.5)	610 (6.1)
Has a patient presented to you the results of a genome-wide scan fat any time in the past 6 months?	156 (4.0)	365 (3.6)
What level of evidence is important/very important to you in consideration of ordering a pharmacogenomic test?		
FDA approval or recommendation	3,051 (76.8)	7,265 (72.9)
Physician specialty guideline	3,191 (80.7)	8,005 (80.4)
Scientific journal publication	2,924 (73.9)	7,487 (75.2)
Recommendation or experience of thought leaders or respected colleagues	2,920 (73.9)	7,463 (75.1)

### 2.3. Factors Associated with Drug Label Use: Univariate Analysis

Compared to physicians who did not report obtaining pharmacogenomic information from drug labels, those that did were significantly older and of greater tenure in their practice careers (Table 3). They were more likely to have obtained a medical degree other than MD or DO and, consistent with this, were also more likely to have attended an ex-US medical school. Differences in practice characteristics were seen, as more were employed in private practice, and had a greater density of Medicare-insured patients.

**Table 3:** Physician characteristics associated with use of drug labels for pharmacogenomics information.

Characteristic	Obtain PGx Info From FDA Label; N = 4054 N (%)	Do Not Obtain PGx Info From FDA Label; N = 6249 N (%)	*p* value
Age (years)			<0.001
20–29	16 (0.4)	20 (0.3)	
30–39	386 (9.6)	699 (11.3)	
40–49	755 (18.8)	1,398 (22.5)	
50–59	1,372 (34.1)	2,138 (34.5)	
60–69	996 (24.8)	1,434 (23.1)	
≥70	499 (12.4)	514 (8.3)	
Medical degree			0.0042
MD	3,524 (88.1)	5,516 (89.2)	
DO	335 (8.4)	517 (8.4)	
Other	141 (3.5)	149 (2.4)	
Years since medical school graduation			<0.0001
<14	681 (17.0)	1,244 (20.1)	
15–29	1,628 (40.7)	2,679 (43.2)	
30+	1,695 (42.3)	2,274 (36.7)	
Location of medical school attended			<0.0001
United States	3,075 (76.8)	5,180 (83.7)	
Europe	156 (3.9)	187 (3.0)	
Other	772 (19.3)	823 (13.3)	
Primary employer			0.0214
Federal/state/military/Veterans Administration	43 (1.1)	76 (1.2)	
Hospital/HMO/other health insurer	296 (7.4)	547 (8.9)	
Medical school/university	100 (2.5)	175 (2.8)	
Private practice/self-employed	3,445 (86.3)	5,166 (83.8)	
Other	110 (2.8)	201 (3.3)	
Primary insurance carrier for majority of patients			0.0032
Medicaid	307 (8.1)	471 (8.0)	
Medicare	1,222 (32.1)	1,691 (28.6)	
Military/Tricare	11 (0.3)	13 (0.2)	
Private insurance	2,118 (55.6)	3,436 (58.2)	
Veterans Administration	6 (0.2)	14 (0.2)	
None	145 (3.8)	281 (4.8)	

Key differences related specifically to pharmacogenomics were also observed, and were quite consistent with those observed from the entire survey sample (Table 4). Physicians that reported obtaining pharmacogenomic information from drug labels had significantly greater prior education and felt adequately informed on pharmacogenomics. Adoption of pharmacogenomic testing in practice was higher in this group, with a relative increase in recent testing experience of 18%, and 36% for those who anticipated testing in the next six months. More often did these physicians report that their patients had benefitted in some manner from pharmacogenomic testing in practice—primarily through improved drug effectiveness or reduced toxicity, improved patient understanding of their disease or therapy, and to a lesser extent, by improving patient adherence to therapy. There was a very strong association with reporting that they “rely” on FDA-approved labeling for pharmacogenomic testing information, with 75% answering yes to both questions, and further with judgment that FDA approval or recommendation for testing was a very important/important level of evidence when considering ordering a pharmacogenomic test. In addition to obtaining testing information from drug labels, these physicians were also significantly more likely to obtain this information from the Internet, a genetic testing laboratory, or a colleague/other physician. 

**Table 4:** Physician knowledge, perception, and practices relating to pharmacogenomics associated with use of drug labels for pharmacogenomics information.

Survey question	Obtain PGx Info From FDA Label; N = 4054 N (%)	Do Not Obtain PGx Info From FDA Label; N = 6249 N (%)	*p* value
Was pharmacogenomics instruction included:			
In your graduate medical education curriculum?	707 (18.0)	758 (12.3)	<0.0001
In your postgraduate medical education?	926 (26.9)	1,093 (20.5)	<0.0001
Do you believe that a patient’s genetic profile may influence his/her response to drug therapy?	3,920 (98.0)	5,950 (97.3)	0.0315
Do you feel that you are adequately informed about the availability of genetic testing and its application in the context of drug therapy?	477(11.9)	571(9.2)	<0.0001
At any time in the past 6 months, have you ordered or recommended a pharmacogenomic test?	611 (15.2)	708 (12.9)	<0.0001
Do you anticipate ordering or recommending a pharmacogenomic test for a patient within the next 6 months?	1221 (31.5)	1371 (23.1)	<0.0001
Where do you obtain information on genetic testing and its application in the context of drug therapy? (select all that apply)			
Internet	1,709 (42.2)	1,810 (29.0)	<0.0001
Genetic testing laboratory	639 (15.8)	738 (11.8)	<0.0001
Colleague/other physician	2,036 (50.2)	2,256 (36.1)	<0.0001
Other	824 (20.3)	2,492 (39.9)	<0.0001
Do you rely on FDA-approved labeling (package inserts) for information regarding genetic testing to predict or improve the response to drugs?	3,016 (75.4)	1,168 (19.2)	<0.0001
Pharmacogenomic tests have benefited your patients by (select all that apply):			
Improving drug effectiveness	490 (12.1)	476 (7.6)	<0.0001
Reducing drug toxicity	542 (13.4)	515 (8.2)	<0.0001
Increasing patients’ understanding of their disease/therapy	407 (10.0)	397 (6.4)	<0.0001
Improving patients’ adherence to therapy	216 (5.3)	202 (3.2)	<0.0001
No tests ordered	2,765 (68.2)	4,449 (71.2)	0.0012
Patients have not benefited	268 (6.6)	556 (8.9)	<0.0001
Do you believe that private, state, and federal health insurers should provide full coverage for pharmacogenomic tests?			0.0005
Always	1,323 (33.5)	1,857 (31.1)	
Sometimes	2,440 (61.7)	3,718 (62.4)	
Never	192 (4.9)	388 (6.5)	
What level of evidence is important/very important to you in consideration of ordering a pharmacogenomic test?			
FDA approval or recommendation	3,051 (76.8)	4,214 (70.3)	<0.0001
Scientific journal publication	2,924 (73.9)	4,563 (76.0)	0.0163
Recommendation or experience of thought leaders or respected colleagues	2,920 (73.9)	4,543 (75.9)	0.0298

Among the physicians surveyed who reported that they “rely” on FDA-approved labeling for pharmacogenomics testing information, general survey responses and the survey items associated with this were patterned very similarly to that reported above for physicians who obtained testing information from drug labels (data not shown).

### 2.4. Predictors of Use of Drug Labeling for Pharmacogenomic Testing Information (Table 5)

The multivariate regression analyses identified several independent predictors of physician use of drug labeling for information on pharmacogenomic testing. Older physicians and physicians that had received pharmacogenomics instruction during postgraduate training were 2.10-fold (*p* = 0.0038) and 1.47-fold (*p* = 0.0002) more likely, respectively, to have reported obtaining pharmacogenomic testing information from drug labeling. Specific aspects relating to alternate information sources and weight of evidence relating to pharmacogenomic testing also were predictive of physician use of drug labeling. Physicians who reported obtaining testing information from drug labeling were also very markedly more likely to “rely” on FDA-approved labeling for information [odds ratio (OR) 14.50 (95% confidence interval, CI, 12.35–17.02); *p* < 0.0001]. These physicians also obtained pharmacogenomic testing information from the internet [OR 1.63 (1.38–1.93); *p* < 0.0001] or from a colleague/other physician [OR 1.54 (1.31–1.81); *p* < 0.0001], but not from other sources [OR 0.56 (0.47–0.67); *p* < 0.0001], and were more likely to consider FDA approval or recommendation a very important or important level of evidence in consideration of ordering a pharmacogenomic test [OR 1.27 (1.06–1.53); *p* = 0.0113]. Physicians who obtained pharmacogenomic testing information from drug labeling were also greater than 2-times more likely to respond that pharmacogenetic testing had provided benefit by increasing their patients’ understanding of their disease and therapy [OR 2.12 (1.32–3.39); *p* = 0.0019].

**Table 5:** Multivariate predictors associated with physician use of drug labels for pharmacogenomics information.

Survey item	Adjusted OR (95% CI)	*p* value
**Physician age**		
30–39 years	1.00	
≥70 years	2.10 (1.46–3.01)	0.0038
**Pharmacogenomic instruction in postgraduate education**		
No	1.00	
Yes	1.47 (1.20–1.81)	0.0002
**Rely on FDA-approved labeling for pharmacogenomics information**		
No	1.00	
Yes	14.50 (12.35–17.02)	<0.0001
**Sources of information on genetic testing and its application in the context of drug therapy**		
Internet		
No	1.00	
Yes	1.63 (1.38–1.93)	<0.0001
Colleague/other physician		
No	1.00	
Yes	1.54 (1.31–1.81)	<0.0001
Other		
No	1.00	
Yes	0.56 (0.47–0.67)	<0.0001
**FDA approval or recommendation is important/very important in consideration of ordering a pharmacogenomic test**		
No	1.00	
Yes	1.27 (1.06–1.53)	0.0113
**Pharmacogenetic tests have benefitted your patients by:**		
Increasing patients’ understanding of theirdisease/therapy		
No	1.00	
Yes	2.12 (1.32–3.39)	0.0019
No tests ordered		
Yes	1.00	
No	0.75 (0.61–0.92)	0.0071

Results are based on multivariate logistic regression, adjusting for all other survey response variables. FDA = Food and Drug Administration, OR = odds ratio, CI = confidence interval.

## 3. Discussion

In this survey of over 10,000 physicians across the United States, we found that 39% reported that they obtained information on pharmacogenomic testing from drug labeling, and in addition, that 42% rely on FDA-approved labeling for this type of information. These figures are consistent with a recent report that 47% of physicians consider medical reference books, such as the Physicians’ Desk Reference, as useful sources of information about pharmaceutical products, and may suggest that pharmacogenomics information is not held to be an exceptional element within labeling [7]. 

In examining predictors of physician use of drug labeling for pharmacogenomic testing information, several important themes emerged. The sole demographic or professional characteristic that was independently associated with use of drug labeling as a source of pharmacogenomic testing information was physician age, and indicated that physicians of longer practice tenure were twice as likely to utilize this information source compare to younger physicians. It may be reasoned that older physicians are more familiar with, and more routinely refer to, drug labeling as an information source, since this dates back to the late 1940s. Physicians who utilized drug labeling as an information source appeared to be generally more likely to be educated in (1.47-fold) and open to seeking pharmacogenomics information from multiple sources, including web-based resources (1.63-fold) and their colleagues (1.54-fold). While inclusion of pharmacogenomics information in drug labeling may not necessarily be a primary driver of test adoption and utilization, these relationships may point to a unique opportunity [8]. As pharmacogenomics content in drug labeling expands, identification of information-seeking physicians and implementation of effective education strategies that may appeal to them could lead to even more use of drug labeling as a source of this information, Further, it also raises the possibility that drug labeling itself could be viewed as a potentially useful educational tool in a receptive physician group. 

Physicians who obtained pharmacogenomic testing information from drug labeling indicated that they also rely on this information. While many sources of drug information are available to physicians, this finding reinforces the perceived authority of drug labeling as a relied-upon data source used in clinical decision-making with respect to pharmacogenomic testing. Likewise, viewing FDA approval or recommendation of a pharmacogenomic test as a very important/important level of evidence in consideration of ordering such a test was predictive of obtaining pharmacogenomic testing information from drug labeling. This, too, would point to the physician perception that regulatory body support for, or provision of, information is authoritative and valuable. 

Lastly, there is a strong indication that obtaining pharmacogenomic testing information from drug labeling is associated with perceived patient benefit of testing, in particular by leading to a greater patient understanding of their disease or therapy. While clearly several translational steps would be required on the part of the physician to affect such a benefit to the patient, the availability of pharmacogenomic testing information in drug labeling and other resources does provide options where physicians can find information that can assist in making this possible.

While there has been some recent controversy over the inclusion of pharmacogenomic information in certain drug labels, witness clopidogrel and warfarin, our survey findings do suggest that it currently is and will most likely continue to be used in some manner. As such, our collective efforts should turn toward ensuring that drug labeling is kept up to date and includes the latest evidence, and that physicians are well-informed on the availability and appropriate application of this information in prescription of these agents. 

Limitations to our survey and our ability to mitigate them have been previously detailed, and relate to survey conduct timing, generalizability, favorable response bias, and validity beyond the responder sample [1]. Key among these, in consideration of the results of this analysis, is that the survey respondents are largely open to and already engaged in pharmacogenomic testing. This would be expected to overestimate the use of drug labeling for pharmacogenomic testing information, and, therefore, the true values may be lower in the broader physician population. It is also important to recognize that there is the potential for disagreement between stated and actual practice patterns, and so what physicians are actually doing would require a different analysis.

The field of pharmacogenomics can be expected to continue to expand in the coming years as pharmacogenomic research extends into new therapeutic areas, and as new drug-companion diagnostic pairings are developed and approved. The findings of this survey analysis indicated that physicians use drug labeling for information on pharmacogenomic testing, that they rely on this information, and that they view FDA-approval or recommendation of testing as a strong level of evidence for consideration. These physicians tended to be of greater practice experience, seekers of information from additional sources, more educated regarding pharmacogenomics, and have noted benefit of testing to their patients. Since these findings clearly demonstrate that physicians engaged in using pharmacogenomics consider drug labeling, they reinforce the need for clear and actionable information in product labeling.

## 4. Methods

### 4.1. Overall Survey

We performed an anonymous, cross-sectional, fax-based survey of United States physician using a 2-page survey questionnaire (see Supplementary) that consisted of 26 questions. Eleven of these were focused on the physicians’ demographic and practice characteristics and 15 assessed physicians’ familiarity, attitudes, and practices with regards to pharmacogenomic testing. The survey was faxed to 225,252 physician offices associated with 397,832 prescribing physicians in September 2008 with a reminder fax sent 2 weeks later. The fax numbers used in targeting this survey came from Medco Health Solutions’ database. Fax communications were unsuccessful in 2,812 (1.2%), which eliminated 9,373 physicians (2.4%) resulting in 222,440 successful faxes to the offices of 388,459 physicians. Physicians responded to the survey by selecting from a limited set of choices for each item. No free-text responses were allowed. No incentive was provided for completion of the survey.

### 4.2. Overall Survey Analysis Approach

Survey responses were included in the analysis if both pages of the survey were returned by fax with at least one completed response per page. Survey questions that were left blank or answered inappropriately were excluded in frequency calculations for that question. Consistency of responses was assessed using a comparison of 6 response pairs that requested similar information. To assess whether the survey respondents were representative of the U.S. physician population, demographic and practice characteristics of the survey respondents were compared to those found in the AMA Physician Masterfile [9].

Some survey responses were collapsed into fewer categories to allow for improved interpretation of results. Number of years since medical school was converted into a 3-level categorical variable: ≤14, 15–29, and ≥30. Primary practice specialty was recoded into a 5-level categorical variable: family/general practice/internal medicine/preventive medicine, non-surgical specialty, surgical specialty, oncology, and other. Primary employer was recoded into a 5-level categorical variable: federal/state/military/Veterans Administration, hospital/HMO/other health insurer, medical school/university, private practice/self-employed, and other. Responses to frequency of ordering or recommending pharmacogenomic tests, responses were stratified into a 2-level variable: 0–1 and ≥2 times/month. Responses to questions assessing the sources of evidence that are important when considering a pharmacogenomic test, responses were restratified into a 3-level categorical variable: “very important/important,” “undecided,” and “very unimportant/unimportant.” For the question “At any time in the past 6 months, have you ordered or recommended a pharmacogenomic test?”, responses were recoded into a 2-level variable: yes (if they had ordered a test) or no (if “none” was selected). Also, for the question “At any time in the past 6 months, have you ordered, recommended, or obtained a genome-wide scan or test?”, responses were recoded into a 2-level variable: yes (if they had ordered a test for one or more categories of patient) or no (if “none” was selected). Responses regarding pharmacogenomics instruction in medical school and postgraduate training were combined into a 2-level variable: yes (if either question was checked “yes”) and no (if both were checked “no”).

### 4.3. Discrete Analysis of Survey Responses Relating to Use of and Reliance on Drug Labeling for Pharmacogenetic Testing Information

Univariate and multivariate analyses were performed that assessed which factors were associated with whether physicians obtained information from drug labels on genetic testing and its application in the context of drug therapy. In our univariate analysis, the dependent variable was the response to the question that asked, “Where do you obtain information on genetic testing and its application in the context of drug therapy? (select all that apply).” This question included five choices which the respondent could use to indicate the sources they use in this context; drug labeling (package insert), internet, genetic testing laboratory, colleague/other physician and other. Specifically, we assessed the factors associated with physicians’ responses to the drug labeling aspect of this question. A 2-level dependent variable was coded based on responses to that question where: 0 = did not indicate that they used the drug label for information on genetic testing and 1 = indicated that they used the drug label for information on genetic testing. An identical univariate analysis was executed for the question, “Do you rely on FDA-approved labeling (package inserts) for information regarding genetic testing to predict or improve the response to drugs?”. However, since this survey item was so strongly related to the item on obtaining information from drug labeling, a separate multivariate analysis was not conducted for this.

In univariate analyses, we assessed statistically significant factors associated with use of the drug label for information on genetic testing and its application in the context of drug therapy using chi square tests. In our multivariate logistic regression analyses, the primary factors associated with use of the drug label to obtain information regarding genetic testing were first identified using stepwise elimination of independent variables. The regression models then evaluated the primary independent variables, adjusting for the secondary variables. Statistical significance in the regression models was evaluated using adjusted odds ratios, 95% confidence intervals, and associated *p*-values. All statistical analyses were performed using SAS version 9.2 (SAS Institute Inc., Cary, NC, USA). All *p*-values were 2-sided; *p* < 0.05 was considered significant. Responses that were collinear with the dependent variables were excluded from the analyses.
